# Progress in structure-based drug development targeting chemokine receptors

**DOI:** 10.3389/fphar.2025.1603950

**Published:** 2025-06-09

**Authors:** Jin Wang, Chen Qu, Peng Xiao, Sijin Liu, Jin-Peng Sun, Yu-Qi Ping

**Affiliations:** ^1^ Medical Science and Technology Innovation Center, Shandong First Medical University & Shandong Academy of Medical Sciences, Jinan, China; ^2^ School of Clinical and Basic Medical Sciences, Shandong First Medical University & Shandong Academy of Medical Sciences, Jinan, China; ^3^ Advanced Medical Research Institute, Cheeloo College of Medicine, Shandong University, Jinan, China

**Keywords:** GPCR, CCRs, cryo-EM, drug discovery, structure-based drug design (SBDD)

## Abstract

As a critical subfamily of G protein-coupled receptors (GPCRs), chemokine receptors (CCRs) play pivotal regulatory roles in immune cell migration, inflammatory modulation, tissue regeneration, and tumor microenvironment (TME) remodeling. By specifically recognizing chemokine ligands, CCRs orchestrate immune cell trafficking and tissue positioning, with functional dysregulation implicated in infectious diseases, autoimmune disorders, neurodegenerative pathologies, and cancer. These receptors thus represent promising therapeutic targets. Recent breakthroughs in cryo-electron microscopy (cryo-EM) and computational chemistry have enabled high-resolution structural analysis and dynamic conformational modeling of CCRs, establishing a robust foundation for structure-based drug design (SBDD). This review synthesizes current advances in CCR biology, structural mechanisms, disease involvement, and targeted drug development, providing theoretical insights and technical frameworks for future research.

## 1 Introduction

G protein-coupled receptors (GPCRs), representing the largest superfamily of cell surface signaling receptors, detect extracellular stimuli and mediate nearly 80% of transmembrane signaling through coupling to heterotrimeric G proteins. GPCRs are ubiquitously expressed across human tissues, with dysregulation linked to cancer, cardiovascular diseases, and neurodegenerative disorders (Stauch and Cherezov, 2018). It is estimated that approximately 34% of approved drugs worldwide target GPCRs, spanning indications including hypertension, allergies, psychiatric disorders, and infections (Scholten et al., 2012; Lin H. et al., 2024; Shen et al., 2024; Hauser et al., 2017; Ji and Tao, 2025). The therapeutic relevance of GPCRs stems not only from their pleiotropic roles but also from their conformational plasticity and pharmacological tractability, making them prime candidates for small molecules, peptides, and antibody-based therapeutics. Recent advances in cryo-EM and computational modeling have unlocked atomic-level insights and dynamic conformational profiling of GPCRs, revolutionizing rational drug design and reinforcing their centrality in modern pharmacology (Yang et al., 2025; Zhou et al., 2024; Shang et al., 2023; Wang et al., 2023; Yang et al., 2021; Ping et al., 2021).

**FIGURE 1:**
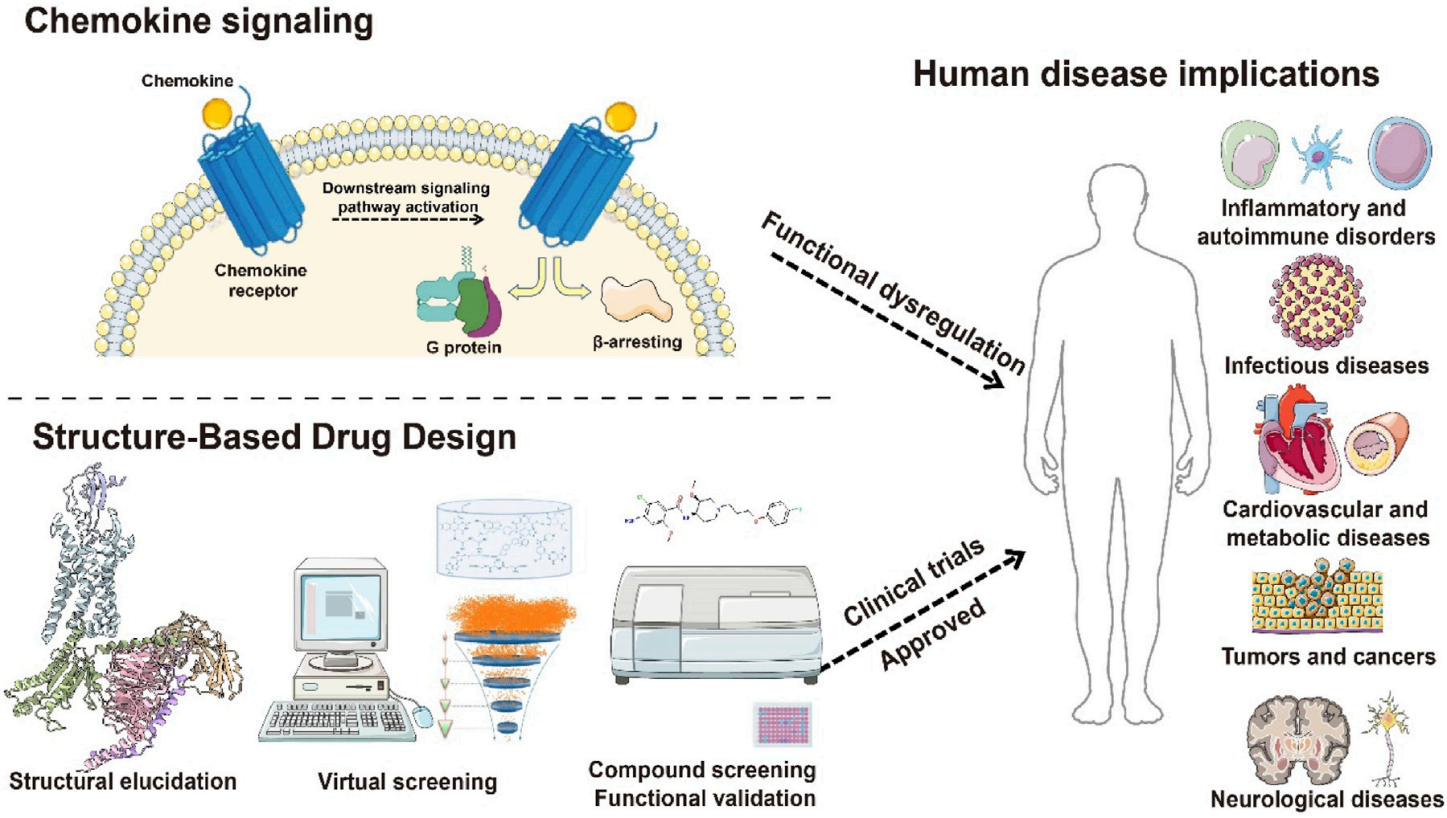
Schematic diagram of structure-based drug development targeting chemokine receptors. This figure illustrates the transmembrane structural features of chemokine receptors, the activation of downstream G protein–and β-arrestin–mediated signaling pathways upon ligand binding, and the functional dysregulation of these receptors in diseases such as inflammation, tumor immunity, and viral infection. It also highlights candidate small-molecule drugs designed based on receptor structural features, providing an integrated framework from receptor structure to function and therapeutic targeting.

Chemokine receptors (CCRs), a subfamily of the GPCR family, belong to the class A (rhodopsin-like) receptors. By recognizing chemokines, CCRs mediate immune cell trafficking and tissue localization, playing pivotal roles in immune surveillance, inflammation, tissue repair, and tumor microenvironment (TME) remodeling. Since the seminal identification of CCR5 and CXCR4 as HIV co-receptors in the 1990s, CCR biology has emerged as a frontier in immunology and therapeutic discovery (Horuk, 2009; Alfano and Poli, 2001). These receptors regulate immune cell function and represent promising targets for immune-related diseases, inflammation, and cancer (Tokunaga et al., 2018; Kazanietz et al., 2019; Sharma, 2010). This review systematically summarizes the biological functions of chemokine-receptor axes, their pathophysiological roles, and recent progress in CCR-targeted drug development, providing a framework for future research (Figure 1).

## 2 Biological basis of chemokines and chemokine receptors

### 2.1 Classification and functions of classical chemokine receptors

#### 2.1.1 Classification and functions of classical chemokine receptors

Chemokines are low-molecular-weight proteins (8–14 kDa) comprising approximately 50 members in mammals, forming the largest cytokine superfamily. Categorized by conserved N-terminal cysteine motifs, they are divided into four subfamilies: CC, CXC, CX3C, and C chemokines (Zlotnik and Yoshie, 2012). The CC subfamily drives monocyte and Th1 cell chemotaxis, central to chronic inflammatory diseases such as rheumatoid arthritis (White et al., 2013). CXC chemokines mediate neutrophil and lymphocyte migration, critical for acute inflammation and tumor metastasis (Anthony et al., 1999; Bikfalvi and Billottet, 2020). The CX3C subfamily contains a single member (CX3CL1), which binds CX3CR1—the sole receptor for CX3C chemokines—to regulate microglia and monocyte migration in the central nervous system (CNS) and atherosclerotic plaques (Imai et al., 1997; Jung et al., 2000). The C subfamily, represented by XCL1 and XCL2, specifically activates XCR1, promoting NK cell-mediated immune surveillance (Bachelerie et al., 2013).

**FIGURE 2:**
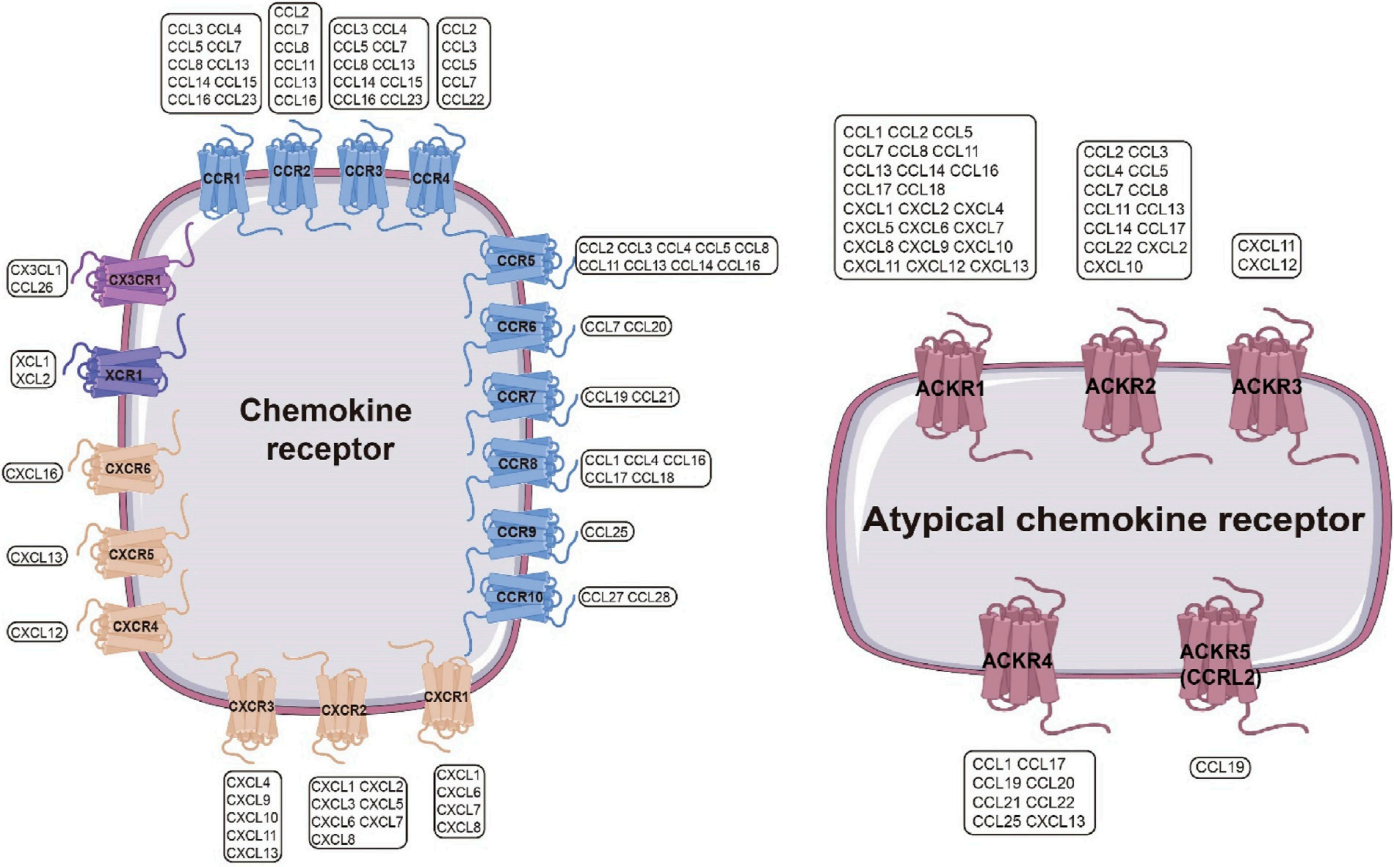
Chemokine Receptor Interaction Network. Left: Chemokine receptors include four families—CCR, CXCR, XCR, and CX3CR—which form a complex interaction network with chemokines. A single chemokine receptor can be activated by multiple chemokines, and a single chemokine can activate multiple chemokine receptors. Blue: CCR; peach: CXCR; gray: XCR; purple: CX3CR.Right: Atypical chemokine receptor family, mainly including ACKR1–ACKR5, can also recognize multiple chemokines. Magenta: ACKR.

Chemokine receptors are classified into four subfamilies (CCR, CXCR, CX3CR, and XCR) based on cysteine motif recognition (Märkl et al., 2022) (Figure 2). The CCR family (CCR1–CCR10) responds to CC chemokines (CCL1–CCL28), with CCR5 recruiting monocytes to inflammatory sites (Lin Y. et al., 2024) and CCR4 directing Th2 cells to allergic lesions (Anderson et al., 2020). The CXCR family (CXCR1–CXCR6) binds CXC chemokines, orchestrating tumor angiogenesis, metastasis, and immune cell recruitment (Horuk, 2009). CX3CR1, exclusively engaged by CX3CL1, is highly expressed in the CNS and atherosclerotic plaques, modulating microglial and monocyte trafficking (Jung et al., 2000). XCR1, the dedicated receptor for XCL1/2, activates NK cell surveillance through phosphorylation-dependent IL-4R signaling (Bachelerie et al., 2013).

The chemokine receptor system transcends its canonical role in immune cell chemotaxis to encompass neuronal regulation, angiogenesis, and TME remodeling. The CXCL12/CXCR4 axis orchestrates hematopoietic stem cell homing to bone marrow niches during embryogenesis. In tumor contexts, malignant cells co-opt this pathway to metastasize to CXCL12-rich organs via Gαi-dependent upregulation of integrin α4β1 and cytoskeletal reorganization (Mempel et al., 2024). The CCL2/CCR2 axis demonstrates context-dependent duality: while driving Ly6C^+^ monocyte recruitment to fuel atherosclerotic plaque progression, it simultaneously polarizes tumor-associated macrophages (TAMs) toward immunosuppressive M2 phenotypes through IL-10 and TGF-β secretion, shaping an immune-evasive niche (de Visser and Joyce, 2023).

Chemokine receptors modulate neurophysiological and disease states, as exemplified by the CX3CR1/CX3CL1 axis fine-tuning microglial activation dynamics in neurodegenerative contexts, directly impacting neuronal viability (Cardona et al., 2006). Angiogenic regulation by chemokines displays microenvironment-specific regulation. The CXCL8/CXCR2 axis emerges as a master regulator of tumor neovascularization, activating ERK1/2 and PI3K pathways in human intestinal microvascular endothelial cells (HIMECs) to drive proliferation, migration, and tubulogenesis. Pharmacologic CXCR2 inhibition potently abrogates these pro-angiogenic effects (Heidemann et al., 2003).

#### 2.1.2 New chemokines and their biological characteristics

Chemokines constitute a master regulatory system for cellular migration and spatial organization. Emerging discoveries reveal novel chemokines with pleiotropic functions extending beyond immune cell trafficking to orchestrate TME dynamics, neuroinflammatory cascades, and tissue regeneration, refining the complexity of chemokine-receptor networks.

##### 2.1.2.1 CX3CL1(Fractalkine)

CX3CL1, the sole CX3C chemokine, features a distinctive domain architecture: an N-terminal chemokine domain fused to a C-terminal transmembrane region. This dual functionality enables CX3CL1 to act as both a soluble chemoattractant and a membrane-anchored adhesion molecule. Binding to CX3CR1 via transmembrane domain (TMD) interactions, CX3CL1 governs monocyte, T cell, and neutrophil migration (Imai et al., 1997).

In neuroinflammation and neurodegeneration, CX3CL1 exerts dual regulatory effects. Soluble CX3CL1-CX3CR1 signaling maintains microglial homeostasis, suppressing hyperactivation and neurotoxic cytokine release (e.g., TNF-α, IL-1β), thereby exerting neuroprotection. Conversely, CX3CR1 deficiency disrupts microglial surveillance, promoting pathological Tau phosphorylation and neuronal degeneration. The axis modulates amyloid-β (Aβ) metabolism: CX3CR1 loss enhances microglial phagocytic activity, reducing fibrillar Aβ plaques, whereas membrane-bound CX3CL1 exacerbates Tau pathology via pro-inflammatory signaling. Adenoviral delivery of soluble CX3CL1 attenuates Tau aggregation and neuronal apoptosis, highlighting therapeutic potential (Cardona et al., 2006; Merino et al., 2016).

In atherosclerosis, CX3CL1 coordinates immune-endothelial crosstalk. Membrane-bound CX3CL1 mediates firm adhesion of monocytes/macrophages to endothelia, while soluble CX3CL1 drives chemotaxis to lesion sites. This dual mechanism fuels inflammatory cell recruitment and pro-inflammatory cytokine release, accelerating endothelial dysfunction and plaque progression (Wu et al., 2025).

Within the TME, CX3CL1-CX3CR1 suppresses antitumor immunity by recruiting regulatory T cells (T_reg_s) and polarizing TAMs. In renal carcinoma, Von Hippel-Lindau tumor suppressor gene (VHL gene) loss upregulates CX3CL1, driving TAM infiltration and establishing immunosuppressive niches (Wolf et al., 2024).

##### 2.1.2.2 XCL1/XCL2

XCL1 and XCL2, prototypic C chemokines, are primarily thymic-derived ligands that specifically engage CD8^+^ T cells and NK cells, critical for immune surveillance and antitumor responses (Kazanietz et al., 2019). XCL1-XCR1 signaling recruits cytotoxic lymphocytes to tumor beds, potentiating tumor cell lysis. These chemokines further modulate antiviral immunity, as evidenced by XCL1 augmenting NK cell-mediated IFN-γ production in cytomegalovirus infection models (Dorner et al., 2004).

##### 2.1.2.3 CXCL16/CXCL17

CXCL16, a dual-function chemokine, exists as soluble and membrane-bound isoforms, expressed broadly across dendritic cells, monocytes, and B lymphocytes to mediate immune-endothelial adhesion (Bao N. et al., 2023). In cancer, CXCL16 exerts paradoxical roles: via CXCR6 activation, it fuels tumor proliferation, invasion, and VEGF/MMP-driven angiogenesis, yet orchestrates antitumor immunity by recruiting NKT cells. Concomitant T_reg_ infiltration, however, may foster immune evasion. CXCL16 further remodels the TME by reprogramming TAMs, Cancer-Associated Fibroblasts (CAFs), and Myeloid-Derived Suppressor Cells (MDSCs), highlighting its therapeutic duality (Korbecki et al., 2021).

CXCL17, a mucosa-enriched chemokine, binds CXCR8 (GPR35) to exhibit context-dependent oncogenicity. It drives tumor progression via TAM recruitment and neovascularization, yet paradoxically primes antitumor immunity. Elevated CXCL17 correlates with clinical outcomes, positioning it as a prognostic biomarker (Gowhari Shabgah et al., 2022).

##### 2.1.2.4 CCL28

CCL28, a multifunctional CC chemokine, binds CCR3/CCR10 to coordinate mucosal immunity and TME dynamics. Beyond broad-spectrum antimicrobial activity against bacteria/fungi, it recruits adaptive immune cells to mucosal interfaces (Mohan et al., 2017). In tumors, CCL28 displays functional dichotomy: enhancing cytotoxic lymphocyte infiltration while facilitating immune suppression via T_reg_/CAF/MDSC recruitment. Mechanistically, the CCL28-CCR10 axis activates PI3K/AKT and MAPK/ERK cascades, propelling tumor growth and metastasis (Mergia Terefe et al., 2022).

### 2.2 Signal transduction mechanisms of chemokine receptors

#### 2.2.1 Classical G protein signaling pathways

**FIGURE 3:**
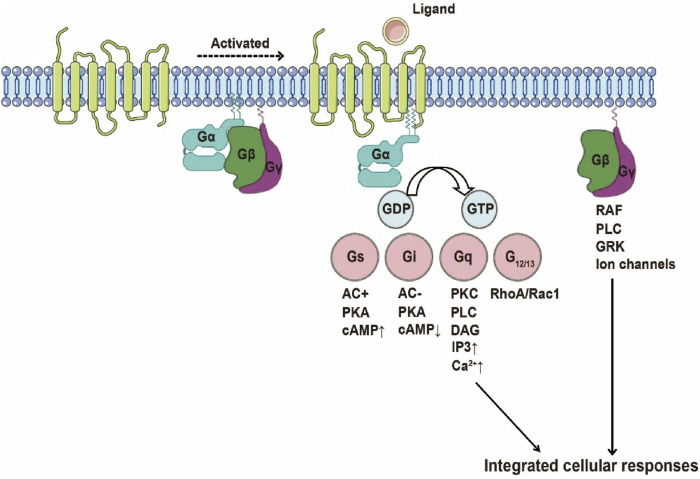
Schematic Illustration of GPCR Activation. Upon ligand binding, the receptor undergoes a conformational change into a pre-activated state, coupled with the G protein heterotrimer. The exchange of GDP for GTP on the G protein α subunit triggers G protein dissociation, leading to the activation of various G protein-mediated signaling pathways, including Gs, Gi, Gq, and G12/13 pathways.

GPCRs represent the largest superfamily of cell surface signaling receptors and play a crucial role in regulating a wide range of physiological processes. As an important subclass of GPCRs, chemokine receptors primarily transduce extracellular chemokine gradient signals into intracellular responses through G protein-dependent pathways, thereby mediating directed migration of target cells and homing of immune cells. Upon chemokine binding, the receptor undergoes a conformational change that activates heterotrimeric G proteins by facilitating the exchange of GDP for GTP on the Gα subunit and promoting dissociation from the Gβγ dimer. The resulting Gα-GTP and free Gβγ subunits interact with various downstream effectors, initiating a series of signaling cascades that lead to cytoskeletal rearrangements and directional migration (Eichel and von Zastrow, 2018) (Figure 3). The classification of chemokines and their receptors is closely linked to the specificity of their downstream signaling and biological functions, especially in regulating immune cell positioning and response dynamics.

G proteins are categorized by α-subunit homology: Gs, Gi, Gq, and G12/13. Gαs/i subunits modulate adenylate cyclase (AC) activity, governing cyclic AMP (cAMP) dynamics. Reduced cAMP levels dampen protein kinase A (PKA) signaling, impacting migration, proliferation, and transcriptional regulation. In CXCR2 signaling, Gαi-GTP suppresses cAMP-PKA pathways to drive tumor invasion (Liu et al., 2022). Gα12/13 activates Rho GTPases (RhoA/Rac1/Cdc42), remodeling cytoskeletal architecture and cell polarity—critical for metastatic dissemination. CXCR4-driven Gα13-RhoA activation exemplifies this pro-metastatic axis (Tan et al., 2006). Gαq stimulates phospholipase Cβ (PLCβ), cleaving PIP2 into IP3 and DAG. IP3 mobilizes intracellular Ca^2+^, while DAG activates PKC-MAPK cascades to control proliferation/differentiation. CCR2a/b receptors leverage Gq-PLCβ signaling and Rho-SRF transcriptional programs to orchestrate migration and inflammation (Vat et al., 2016).

The Gβγ dimer directly activates PLCβ to amplify IP3/DAG production, coupling Ca^2+^ flux and PKC activation to cytoskeletal dynamics. Concurrently, Gβγ engages PI3K to generate PIP3, activating Akt survival pathways and motility programs (Jiang et al., 2022).

Chemokine signaling is silenced via Gα GTPase activity: GTP hydrolysis regenerates the inactive Gαβγ heterotrimer. GRK-mediated phosphorylation and β-arrestin recruitment drive receptor internalization and desensitization. Notably, β-arrestin scaffolds MAPK activation, extending signaling beyond canonical G protein pathways (Lefkowitz and Shenoy, 2005; Reiter et al., 2012).

#### 2.2.2 Non-canonical signaling pathways in chemokine biology

Chemokine-receptor axes signal via both classical G protein cascades and alternative pathways, regulating migration, activation, proliferation, and survival through divergent mechanisms.

##### 2.2.2.1 Receptor tyrosine kinase (RTK) crosstalk

CXCR4 primarily engages Gi signaling upon CXCL12 binding. Pharmacologic CXCL12-CXCR4 blockade (e.g., plerixafor) triggers compensatory survival programs via RTK activation, notably PDGFRB, sustaining proliferation and evading apoptosis (Berning et al., 2018).

##### 2.2.2.2 PI3K/AKT axis

The CXCL12-CXCR4 axis activates PI3K/AKT in colon cancer, driving proliferation and metastasis through PTEN suppression. Concurrently, CXCL10-CXCR3 signaling induces AKT-dependent MMP-2/9 upregulation, facilitating ECM proteolysis and gastric cancer dissemination (Ma et al., 2019; Zhou et al., 2016).

##### 2.2.2.3 MAPK/ERK dynamics

CXCR2 activates ERK/p38 MAPK to orchestrate fibroblast chemotaxis. Atherosclerosis-Associated Endothelial cell-Specific Immunogenic Sequence-1 peptide (AESIS-1 peptide) potentiates wound healing by amplifying ERK/MAPK-driven CXCR2 expression, enhancing chemotactic responsiveness (Park et al., 2024).

##### 2.2.2.4 NF-κB inflammatory circuitry

CXCR1 propagates myocardial ischemia/reperfusion injury via NF-κB activation, upregulating COX-2, ICAM-1, and VCAM-1 to amplify leukocyte infiltration. CXCR1 inhibition dampens NF-κB activity, attenuating inflammation and tissue damage (Xi et al., 2022).

##### 2.2.2.5 JAK/STAT-HIV nexus

CCR5 sustains CD4^+^ T cell expression through JAK/STAT signaling, enabling R5-tropic HIV entry. JAK/STAT inhibition reduces CCR5 surface density, impairing viral infectivity and highlighting therapeutic potential (Wang et al., 2024).

### 2.3 Conformational changes of chemokine receptors mediating their physiological functions

Chemokine receptors, pivotal members of the GPCR superfamily, orchestrate immune cell trafficking, inflammatory modulation, and TME remodeling. Their dysregulated signaling is intimately linked to autoimmune diseases, infections, and cancer progression, positioning them as critical therapeutic targets. Recent breakthroughs in cryo-EM have unlocked high-resolution structures of these receptors, revealing molecular details of ligand engagement, activation dynamics, and disease-associated conformational states (Table 1).

**TABLE 1:** Resolved structures of chemokine receptors.

GPCR	Resolved structure	PDB code	Method	Resolution	Release date	References
CCR1	Apo CCR1	7VL8	cryo-EM	2.90 Å	2022	Shao et al. (2022a)
	CCL15(26/27-92)-CCR1	7VL9/7VLA	cryo-EM	2.60 Å/2.70 Å		
CCR2	CCL2–CCR2–Gi	7XA3	cryo-EM	2.90 Å	2022	Shao et al. (2022b)
CCR3	CCR3-Gi	7X9Y	cryo-EM	3.10 Å	2022	Shao et al. (2022b)
CCR5	CCR5-Maraviroc	4MBS	X-RAY	2.71 Å	2013	Tan et al. (2013)
	(5P7)CCL5-CCR5	5UIW	X-RAY	2.20 Å	2017	Zheng et al. (2017)
	compound 21-CCR5	6AKX	X-RAY	2.80 Å	2018	Peng et al. (2018)
	compound 34-CCR5	6AKY	X-RAY	2.80 Å		
	gp120-CD4-CCR5	6MEO	cryo-EM	3.90 Å	2019	Shaik et al. (2019)
	CCR5-Gi	7F1S	cryo-EM	2.80 Å	2021	Zhang et al. (2021)
	MIP-1a-CCR5	7F1T	X-RAY	2.60 Å		
	MIP.1a -CCR5-Gai	7F1Q	cryo-EM	2.90 Å		
	RANTES-CCR5-Gi	7F1R	cryo-EM	3.00 Å		
	CCL5-CCR5-Gi-Fab16	7O7F	cryo-EM	3.15 Å	2021	Isaikina et al. (2021)
CCR6	CCL20-CCR6-Gao	6WWZ	cryo-EM	3.34 Å	2020	Wasilko et al. (2020)
	SQA1-OXM2-CCR6	9D3E	cryo-EM	3.02 Å	2024	Wasilko et al. (2024)
	SQA1-OXM2-CCR6	9D3G	cryo-EM	3.26 Å		
CCR7	Cmp2105-CCR7	6QZH	X-RAY	2.10 Å	2019	Jaeger et al. (2019)
CCR8	CCR8-Fab1	8TLM	cryo-EM	2.90 Å	2023	Sun et al. (2023)
	CCL1-CCR8-Gi	8U1U	cryo-EM	3.10 Å		
	Apo CCR8-Gi	8KFZ	cryo-EM	3.30 Å	2024	Jiang et al. (2024)
	LMD-009-CCR8-Gi	8KFY	cryo-EM	3.06 Å		
	ZK 756326-CCR8-Gi	8KFX	cryo-EM	2.96 Å		
CCR9	Vercirnon-CCR9	5LWE	X-RAY	2.80 Å	2016	Oswald et al. (2016)
CXCR1	CXCR1	2LNL	SOLID-STATE NMR	None	2012	Park et al. (2012)
	CXCL8-CXCR1-Gi	8IC0	cryo-EM	3.41 Å	2023	Ishimoto et al. (2023)
CXCR2	CXCR2 inactive	6LFL	X-RAY	3.20 Å	2020	Liu et al. (2020)
	CXCL8 dimer/monomer-CXCR2-Gi	6LFM/6LFO	cryo-EM	3.50 Å/3.40 Å		
	CXCL1-CXCR2	8XWA	cryo-EM	3.48 Å	2025	To be published
	CXCL2-CXCR2	8XVU	cryo-EM	3.09 Å		
	CXCL3-CXCR2	8XWF	cryo-EM	3.65 Å		
	CXCL5-CXCR2	8XWS	cryo-EM	3.06 Å		
	CXCL6-CXCR2	8XWM	cryo-EM	3.71 Å		
	CXCL8-CXCR2	8XWN	cryo-EM	3.29 Å		
	CXCL1-CXCR2-Go	8XWV	cryo-EM	3.07 Å		
	CXCL2-CXCR2-Go	8XXH	cryo-EM	2.80 Å		
	CXCL3-CXCR2-Go	8XX3	cryo-EM	3.38 Å		
	CXCL5-CXCR2-Go	8XX7	cryo-EM	3.32 Å		
	CXCL6-CXCR2-Go	8XXR	cryo-EM	3.17 Å		
	CXCL8-CXCR2-Go	8XX6	cryo-EM	2.99 Å		
CXCR3	AMG487-CXCR3-Nb6	8K2W	cryo-EM	3.00 Å	2023	Jiao et al. (2023)
	CXCL10-CXCR3-DNGi	8K2X	cryo-EM	3.20 Å		
	CXCL11-CXCR3-DNGi	8HNK	cryo-EM	3.01 Å	2024	Jiao et al. (2024)
	PS372424-CXCR3-DNGi	8HNL	cryo-EM	2.98 Å		
	VUF11222-CXCR3-DNGi	8HNM	cryo-EM	2.94 Å		
	SCH546738-CXCR3-Nb6	8HNN	cryo-EM	3.60 Å		
	CXCR3-Go	8XXZ	cryo-EM	3.30 Å	2025	To be published
	VUF10661-CXCR3	8XYI	cryo-EM	3.16 Å		
	VUF11418-CXCR3	8Y0H	cryo-EM	3.53 Å		
	VUF10661-CXCR3-Go	8XYK	cryo-EM	3.03 Å		
	VUF11418-CXCR3-Go	8Y0N	cryo-EM	3.07 Å		
CXCR4	IT1t-CXCR4	3ODU	X-RAY	2.50 Å	2010	Wu et al. (2010)
	CVX15-CXCR4	3OE0	X-RAY	2.90 Å		
	vMIP-II-CXCR4	4RWS	X-RAY	3.10 Å	2015	Qin et al. (2015)
	CXCL12-CXCR4-Gi	8K3Z	cryo-EM	2.81 Å	2024	Liu et al. (2024)
	CXCR4-Gi	8U4N	cryo-EM	2.72 Å	2025	Saotome et al. (2025)
	CXCL12-CXCR4-Gi	8U4O	cryo-EM	3.29 Å		
	AMD3100-CXCR4-Gi	8U4P	cryo-EM	3.15 Å		
	REGN7663 Fab-CXCR4-Gi	8U4Q	cryo-EM	3.36 Å		
	REGN7663-CXCR4-Fab	8U4R	cryo-EM	3.10 Å		
	REGN7663-CXCR4-Fab trimer	8U4S	cryo-EM	3.35 Å		
	HF51116-CXCR4	8ZPL	cryo-EM	3.01 Å	2025	To be published
	AMD070-CXCR4	8ZPM	cryo-EM	3.20 Å		
	AMD3100-CXCR4	8ZPN	cryo-EM	3.31 Å		
CXCR7	CID25-ACKR3-CXCL12(WT)-CID24	7SK3/7SK4/7SK5	cryo-EM	3.80 Å/3.30 Å/4.00 Å	2022	Yen et al. (2022)
	CXCL12-ACKR3-CCX662-Fab	7SK6/7SK7/7SK8/7SK9	cryo-EM	4.00 Å/3.30 Å/3.30 Å/3.70 Å		
CXCR8	Lodoxamide-GPR35-G13	8H8J	cryo-EM	3.20 Å	2022	Duan et al. (2022)
CX3CR1	Apo CX3CR1-Gi	7XBW	cryo-EM	2.80 Å	2022	Lu et al. (2022)
CX3CR1	CX3CL1-CX3CR1-Gi	7XBX	cryo-EM	3.40 Å	2022	Lu et al. (2022)
XCR1	XCL1-XCR1-Gi	9AST	cryo-EM	3.07 Å	2024	Zhang et al. (2024)

Chemokine receptors leverage a conserved seven-transmembrane (TM1–7) helical bundle coupled with dynamic extracellular/intracellular loop (ECL/ICL) remodeling to achieve ligand specificity and signal transduction. Ligand binding follows a two-site recognition paradigm: the chemokine core engages the receptor N-terminus and ECLs via chemokine recognition site 1 (CRS1), while its N-terminal domain inserts into the TMD at CRS2, triggering activation. Structural studies of the CXCR4-CXCL12 complex illustrate this mechanism: CXCL12’s N-terminus penetrates the CRS2 pocket through salt bridges (D97/E288) and hydrophobic interactions, inducing a 10-Å TM6 outward shift to expose the G protein-binding interface. This conserved activation mechanism underpins receptor selectivity (e.g., CXCR4-CXCL12 exclusivity) and functional plasticity, enabling biased signaling toward G protein or β-arrestin pathways (Liu et al., 2024).

Chemokine receptor activation mechanisms rely critically on conformational dynamics within transmembrane helices and conserved functional motifs. For instance, CCR5 activation involves the toggle switch residues Y251^6.51^ and W248^6.48^, propagating structural changes through DRY, PIF, and NPxxY motifs to drive G protein coupling (Zhang et al., 2021). Structural studies of the CCR6/CCL20 complex reveal an atypical activation pathway: CCL20 binds superficially via a salt bridge with E198, inducing allosteric displacements in TM3, TM4, and TM6 without engaging classical toggle switch residues, illustrating mechanistic diversity in GPCR signaling (Wasilko et al., 2020). Constitutive activation phenomena, such as W86^2.60^ conformational shifts in CCR5, demonstrate ligand-independent signaling through intrinsic helix dynamics, potentially contributing to chronic inflammation and autoimmune pathogenesis.

G protein coupling modes among chemokine receptors exhibit both conservation and divergence. In Gαi-coupled systems, CXCR2 utilizes its α5 helix to interact with TM3, TM5, TM6, ICL2, and ICL3, where hydrophobic residues (e.g., L353, L348) anchor into CXCR2’s hydrophobic pocket (Liu et al., 2020). Contrastingly, CCR5 employs ICL2 and ICL3 synergistically for Gαi engagement, with the α5 helix inserting into the intracellular region and stabilizing interactions through analogous hydrophobic contacts, while ICL3 assumes greater functional importance compared to CXCR2 (55). CX3CR1 displays distinct coupling architecture: minimal TM6 outward movement creates a constrained G protein-binding pocket, compensated by TM7 and H8 displacements. This receptor forms an extended interaction network involving TM1–TM7, ICL2, and ICL3, with a cholesterol-binding site stabilizing helix VI to modulate interface formation (Lu et al., 2022).

Structural elucidation of chemokine receptors has revolutionized our understanding of disease pathogenesis and therapeutic discovery. High-resolution structural studies clarified HIV-1’s co-receptor hijacking mechanism: viral gp120 structurally mimics chemokine engagement, with its V3 loop penetrating the CRS2 pocket through charge complementarity with receptor N-termini. Maraviroc sterically hinders gp120 binding by occupying the transmembrane allosteric pocket, unveiling precise inhibition mechanisms (Tan et al., 2013). The anti-CXCR4 antibody REGN7663 exemplifies structure-guided precision medicine, competing for extracellular binding via CDR-H3 loop interactions. In oncology, receptor oligomerization emerges as a novel therapeutic axis—cholesterol-stabilized CXCR4 tetramers constrain conformational flexibility to impair G protein coupling, proposing oligomeric state modulation for metastasis intervention (Saotome et al., 2025).

Collectively, chemokine receptor structural biology has decoded fundamental principles of ligand recognition, activation dynamics, and signal propagation while illuminating pathophysiological mechanisms in immunity, infection, and cancer. Integration of crystallography and cryo-EM captures receptor conformational landscapes across functional states, enabling rational design of allosteric modulators, biased agonists, and therapeutic antibodies. Future advances in receptor-complex structural determination coupled with AI-driven molecular engineering promise pathway-selective therapeutics targeting specific receptor subtypes, propelling innovation in treating inflammation, autoimmunity, and malignancies.

### 2.4 Interaction models between chemokines and chemokine receptors

**FIGURE 4:**
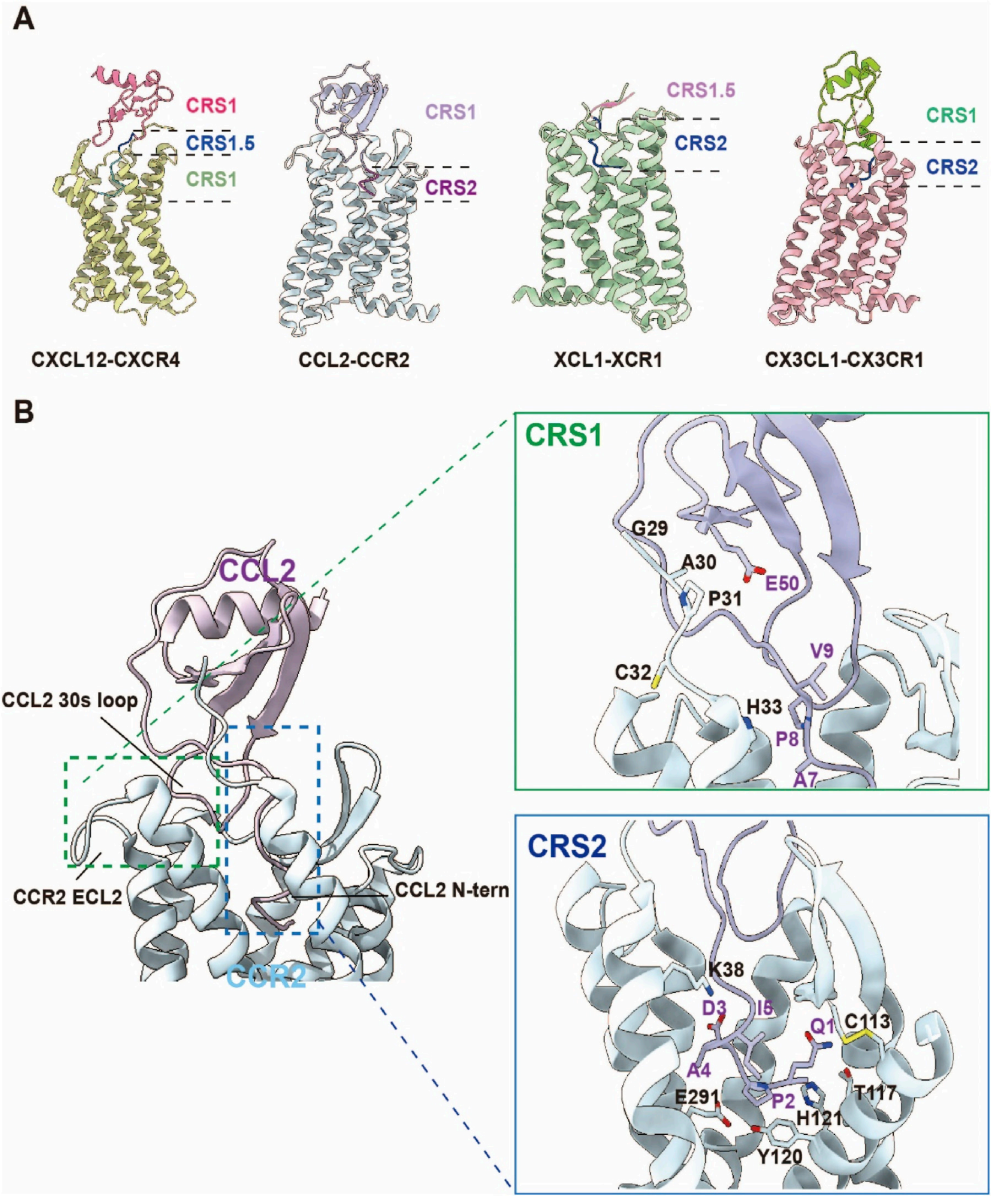
Schematic Diagram of the Chemokine “Two-Site Model”. **(A)** The recognition of chemokines by the four chemokine receptor families—CCR, CXCR, CX3CR, and XCR—follows the “two-site model.” CRS1 primarily involves interactions between the globular core region of the chemokine and the N-terminal domain (N-term) and extracellular loops (ECLs) of the receptor. CRS1.5 plays a crucial bridging role in ligand recognition and receptor activation, ensuring the correct insertion of the chemokine N-terminus into the transmembrane binding pocket. CRS2 is mainly responsible for the insertion of the chemokine N-terminus into the receptor’s transmembrane domain, forming a structural binding pocket. (CXCL12-CXCR4 complex PDB:8K3Z; CCL2-CCR2 complex PDB:7XA3; XCL1-CXR1 complex PDB:9AST; CX3CL1-CX3CR1 complex PDB:7XBX). **(B)** Structural overview of CCR2 in complex with its endogenous ligand CCL2. Left: The orthosteric chemokine-binding pocket of CCR2 and the overall architecture of the CCR2–CCL2 complex. Right: Detailed interactions between CCR2 and CCL2 at the chemokine recognition sites CRS1 and CRS2.

The “Two-Site Model” of chemokine-receptor interactions provides a structural paradigm for understanding ligand recognition and activation mechanisms (Figure 4). In this framework, chemokine binding occurs through two discrete sites that cooperatively induce receptor conformational changes and initiate downstream signaling.

CRS1 mediates initial high-affinity engagement through interactions between the chemokine’s globular core and the receptor’s N-terminus/ECLs. The chemokine core, stabilized by conserved disulfide bonds, adopts a compact tertiary structure whose N-loop and 40s-loop form polar and hydrophobic contacts with receptor residues. In the CCR2-CCL2 complex, CCL2’s N-loop and 40s-loop establish hydrogen bonds and salt bridges (e.g., Y49, D52) with CCR2’s N-terminus and ECL2, anchoring the primary binding interface while priming N-terminal insertion into the transmembrane pocket (Shao et al., 2022b). Similarly, CX3CL1’s globular core engages CX3CR1’s N-terminus and ECL2 via disulfide-mediated interactions (C28, C109), reinforcing CRS1 binding stability (Wasilko et al., 2020).

CRS2 involves N-terminal penetration of the chemokine into the receptor’s TMD, forming an orthosteric activation pocket. The chemokine’s N-terminus (5–10 residues) inserts into TMD helices, establishing polar and hydrophobic networks that trigger conformational rearrangements. In CCR8-CCL1 complexes, CCL1’s R24/K25 residues penetrate the TMD, forming hydrogen bonds and salt bridges with TM2/TM3/TM7 residues (Y113, D284) (Sun et al., 2023). XCL1’s K1/R2 similarly interact with XCR1’s TM3/TM5/TM7 (Y241, L245), stabilizing the active conformation and exposing the G protein-binding interface (Zhang et al., 2024).

The two-site model hinges on cooperative engagement of CRS1 and CRS2, where CRS1 primes structural transitions that enable chemokine N-terminal insertion into CRS2, triggering receptor activation. This stepwise mechanism ensures high-affinity ligand-receptor binding while enabling signaling specificity through precise conformational control. In the CCR6-CCL20 complex, CCL20’s globular core binds CCR6’s N-terminus and ECL2 via CRS1, while its N-terminus inserts into the TMD through CRS2, forming interactions with TM2/TM3/TM7 residues (E198, Y291). This dual-site architecture explains both ligand-receptor specificity and activation mechanics (Wasilko et al., 2020).

Beyond explaining binding affinity, the two-site model illuminates receptor activation principles and signal diversification mechanisms. By orchestrating conformational precision, chemokines gate specific downstream pathways, reflecting functional plasticity in ligand recognition. This framework also guides therapeutic design: maraviroc mimics chemokine binding to occupy CCR5’s CRS2, sterically blocking HIV gp120 engagement (Tan et al., 2013), while AMD3100 antagonizes CXCR4 by occluding CXCL12 binding at CRS2, inhibiting oncogenic signaling (Saotome et al., 2025).

Building on the two-site model, a more refined “three-step model” has been proposed: chemokines initially form a low-affinity, non-specific interaction with the receptor’s N-terminus through the N-loop/β3 region; subsequently, the N-terminus of the chemokine engages in a rate-limiting interaction with the receptor’s second binding site, achieving high-affinity and specific binding; finally, the receptor undergoes conformational rearrangement and activation (Sanchez et al., 2019). This model provides a more precise description of the complex binding and activation process between chemokines and their receptors.

In summary, the two-site model deciphers molecular logic underlying chemokine signaling and disease pathogenesis. Integrating structural and functional insights will accelerate targeted drug discovery, harnessing receptor dynamics for therapies against inflammation, infection, and cancer.

## 3 Roles of chemokine receptors in diseases

### 3.1 Regulation of chemokine receptors in autoimmune diseases

Autoimmune diseases (AIDs) are disorders driven by immune system dysregulation, where self-tolerance is breached, leading to pathogenic attack on host tissues (Basta et al., 2020). Central to this process, chemokine receptors orchestrate immune cell trafficking, directing leukocyte migration to sites of inflammation—a mechanism intricately linked to AID initiation and progression. Beyond migration, chemokines modulate immune cell activation, proliferation, and microenvironment remodeling, amplifying inflammatory cascades. Dysregulated chemokine-receptor axes frequently drive aberrant immune cell infiltration and tissue injury, as exemplified in systemic lupus erythematosus (SLE) and rheumatoid arthritis (RA).

SLE, a multisystem autoimmune disorder, manifests through autoantibody-mediated tissue damage influenced by genetic, environmental, and immune factors (Basta et al., 2020). Notably, chemokine-receptor networks are hyperactivated in SLE, particularly at inflammatory sites. In lupus nephritis (LN), CXCL13-CXCR5 signaling recruits B cells to kidneys, exacerbating renal inflammation, while CXCL9/10-CXCR3 axes drive T cell infiltration (Duan L. et al., 2024). Cutaneous lupus lesions exhibit CXCR3-mediated trafficking of memory/effector T cells via CXCL9-11, perpetuating skin damage. In SLE-associated cardiovascular complications, CXCR3^+^ T cell infiltration into arterial walls accelerates atherosclerosis. Neuropsychiatric SLE (NPSLE) involves CNS-targeted chemokines (e.g., CXCL8, CCL2, CXCL10) that facilitate monocyte and T cell migration, fostering neuroinflammation (Duan L. et al., 2024). Therapeutic targeting of chemokine pathways in SLE holds promise. CXCL13, CXCL12, and CCL2 blockade attenuates renal inflammation in preclinical models, while agents like bindarit show efficacy in early trials (Ble et al., 2011). However, functional redundancy within chemokine systems necessitates multiplex targeting strategies to overcome compensatory pathways.

RA, characterized by synovial hyperplasia and joint destruction, relies on chemokine-guided immune cell infiltration. Synovial T cell recruitment is mediated by CCR4, CCR5, CXCR3, CXCR4, and CXCR6, while CCR6 specifically directs Th17 cell migration (Murayama et al., 2023). B cell homing and germinal center formation depend on CXCL13-CXCR5 interactions, whereas CXCR1/2 orchestrate neutrophil influx via CXCL8. Beyond cell trafficking, chemokine receptors drive synovial angiogenesis (CXCL12-CXCR4) and bone erosion (CCR2/5-monocyte/macrophage axis) (Murayama et al., 2023). Multifunctional roles of these receptors underscore their potential as therapeutic nodes for halting RA progression.

### 3.2 Chemokine receptors in infectious diseases

HIV (Human Immunodeficiency Virus) subverts host immunity by selectively depleting CD4^+^ T cells, with chemokine receptors serving pivotal roles in viral entry. CXCR4 and CCR5 function as principal co-receptors for T-tropic and macrophage-tropic HIV-1 strains, respectively. During early infection, CCR5-dependent viral entry into macrophages and resting T cells correlates with indolent disease progression. Virological evolution toward CXCR4 tropism enables activated T cell infection, driving rapid CD4^+^ depletion and clinical deterioration (Kalinkovich et al., 1999).

The CCR5Δ32 mutation confers natural resistance to HIV-1, inspiring therapeutic CCR5 antagonists like maraviroc for clinical use (Venuti et al., 2017). CXCR4-targeted strategies face translational challenges due to its essential physiological roles in hematopoiesis and immunity. While CXCR4 inhibitors (e.g., AMD3100) mobilize hematopoietic stem cells (Huang et al., 2021), systemic blockade risks disrupting homeostatic functions. Precision targeting of CXCR4-HIV interactions may emerge through mechanism-based drug design, expanding therapeutic options.

Chemokine receptors orchestrate immune cell dynamics across inflammation stages. CXCR2 directs neutrophil chemotaxis via IL-8/CXCL8 sensing, establishing frontline antimicrobial defense. Concurrently, CCR2-CCL2 signaling mobilizes monocytes from bone marrow to inflamed tissues, differentiating into macrophages/DCs for pathogen clearance and tissue remodeling.

Inflammatory resolution involves chemokine receptor-mediated immune cell reprogramming. CCR7 upregulation licenses antigen-laden DCs to migrate toward lymphoid CCL19/CCL21 gradients, bridging innate and adaptive immunity through T cell priming. This spatiotemporal regulation enhances antigen-specific responses while preventing immunopathology.

Chemokine receptors operate within dynamic signaling networks: CXCR4/CCR5 coregulate immune recruitment, with expression levels mirroring inflammatory phase transitions. Receptor homo-/heterodimerization further amplifies chemosensitivity, enabling nuanced immune regulation within complex microenvironments (Chen et al., 2018).

### 3.3 Chemokine receptors as orchestrators of tumor immune dynamics

Chemokine receptors critically regulate TME dynamics by mediating crosstalk between immune and tumor cells, driving tumor progression, metastasis, and immune evasion. The CXCR4-CXCL12 axis is pivotal for tumor cell migration and pre-metastatic niche formation in bone marrow and lymph nodes, while chemokine receptor-mediated recruitment of TAMs and MDSCs establishes an immunosuppressive microenvironment that subverts immune surveillance (Bian et al., 2019).

These receptors orchestrate immune cell trafficking from the vasculature into tumors and spatially organize their distribution, enabling functional interactions with stromal and malignant cells. CXCR3, via ligands CXCL9/10/11, enhances CD8^+^ T cell and NK cell infiltration and synergizes with antigen-presenting cells to amplify antitumor immunity. CXCR6-CXCL16 signaling sustains effector T cell survival in perivascular niches through crosstalk with CXCL16^+^ dendritic cells, whereas CCR4/CCR8 facilitate T_reg_ accumulation to suppress immune responses (Mempel et al., 2024). Dysregulated chemokine receptor activity is intimately linked to immune checkpoint blockade (ICB) resistance. CCR4-dependent T_reg_ infiltration represents a central resistance mechanism, positioning CCR4 antagonists and CXCR4 inhibitors as strategic tools to remodel the TME and restore immunotherapy efficacy. CXCR4 inhibitors block metastasis through multiple mechanisms, including competitive inhibition of CXCL12 binding, prevention of G protein activation, and promotion of receptor internalization. By disrupting ligand-induced CXCR4 signaling, these inhibitors impede downstream pathways involved in cytoskeletal remodeling, adhesion, and migration, thereby limiting tumor cell dissemination and metastatic colonization. Additionally, CXCR4 blockade modulates immune cell positioning within the tumor microenvironment, enhancing immune infiltration and improving responses to immunotherapy (Yi et al., 2024).

Chemokine receptors enable tumor immune escape through multifaceted mechanisms. The CXCL12-CXCR4 axis recruits T_reg_s and MDSCs to inhibit cytotoxic T/NK cell function, while hypoxia and acidosis downregulate CXCR3/CCR5 on NK cells, impairing their migration. Tumor-derived TGF-β suppresses chemokine receptor expression, limiting immune cell infiltration. Concurrently, CCL22-CCR4 and CCL2-CCR2 axes drive immunosuppression via T_reg_/MDSC/TAM recruitment, and CXCL8-CXCR1/2 signaling recruits granulocytic MDSCs/neutrophils to promote angiogenesis and immune suppression. Furthermore, CXCL12-CXCR4 induces regulatory CD8^+^ T cells that inhibit tumor-specific effector T cell activation, culminating in immune escape (Ran et al., 2022).

### 3.4 Dual roles of chemokine receptors in neurological disorders

Chemokine receptors exhibit dualistic roles in neurological pathologies, balancing neuroprotection and exacerbation of damage. CX3CR1 orchestrates microglial dynamics in the CNS, regulating inflammatory tone and synaptic refinement. During neurodevelopment, CX3CR1 guides microglial brain infiltration, maintaining homeostasis and suppressing inflammation to support memory and learning. While CX3CR1 restrains excessive microglial activation under steady-state conditions, it licenses reactive gliosis during inflammation, exhibiting context-dependent neuroprotection or injury (Sheridan and Murphy, 2013). CX3CR1 upregulation in neuroinflammation models fuels pathogenic crosstalk between activated glia and neurons, amplifying inflammatory cascades (Xu et al., 2016).

In neurodegenerative pathologies such as Parkinson’s (PD) and Alzheimer’s (AD) diseases, CX3CR1 modulates microglial responses to neuronal injury. PD models reveal that CX3CR1 loss impairs microglial damage sensing, escalating neuroinflammation. Normally, CX3CR1-CX3CL1 signaling dampens microglial hyperactivation to preserve neural homeostasis; CX3CR1 deficiency, however, compromises debris clearance, worsening neuronal loss and inflammatory escalation. Conversely, CX3CR1 overexpression triggers microglial overactivation and pro-inflammatory mediator release, disrupting synaptic integrity and neuronal transmission (Subbarayan et al., 2022). In AD models, microglial CX3CR1 ablation mitigates neuronal degeneration, highlighting its disease-stage-specific duality (Fuhrmann et al., 2010).

## 4 Advances in structure-based drug development targeting chemokine receptors

### 4.1 Drug design strategies targeting GPCRs

Chemokine receptors play central roles in various pathophysiological processes, including inflammation, immune cell migration, tumor microenvironment remodeling, and viral entry. The interaction between chemokines and their receptors exhibits a pronounced ligand–receptor promiscuity, where a single chemokine can activate multiple receptors, and conversely, a single receptor can be triggered by multiple chemokines. As a result, the development of highly selective ligands at the molecular level remains a major challenge in chemokine-targeted drug discovery. Systematic sequence alignment provides a crucial theoretical basis for understanding the conserved and variable features among chemokine receptor subtypes, thereby informing rational design of selective therapeutics (Supplementary Figure S1).

First, the transmembrane helices (TM1–TM7) of chemokine receptors display a high degree of conservation across receptor families, particularly with respect to structurally and functionally critical motifs. These include the DRY (Asp-Arg-Tyr) motif in TM3, the CWxP motif in TM6, and the NPxxY motif in TM7. These conserved sequences are essential for G protein coupling, conformational changes, and activation, thereby maintaining core signaling functions of GPCRs. The alignment reveals that these motifs are ubiquitously conserved across the CCR, CXCR, XCR, and CX3CR families. Their conservation implies that small-molecule ligands targeting these motifs are likely to interact with multiple receptor subtypes, increasing the risk of off-target effects. Therefore, such regions are more appropriate as structural scaffolds to retain functional integrity rather than as primary targets for pharmacological intervention.

In contrast, the extracellular regions—particularly the second extracellular loop (ECL2) and the N-terminal domain—exhibit substantial sequence variability among different receptors. These domains are directly involved in ligand recognition and binding, and critically influence receptor specificity and affinity. For example, while CCR5, CCR2, and CX3CR1 share similar transmembrane architecture, alignment reveals marked differences in their ECL2 and N-terminal sequences in terms of amino acid length, polarity, charge distribution, and potential glycosylation sites. These differences contribute to distinct ligand-binding microenvironments, which underlie the selective activity of chemokine receptor-targeted agents such as the CCR5 antagonist Maraviroc. Sequence alignment thus helps to identify “selectivity-determining residues,” which serve as precise molecular targets for SBDD.

Moreover, sequence comparison facilitates the identification of receptor subtype-specific structural features, which can guide the development of covalent inhibitors, allosteric modulators, or monoclonal antibody therapeutics. For instance, compared with other CXCR receptors, CX3CR1 contains several non-conserved residues within the TM5–TM7 region, which may form unique hydrophobic or cryptic binding pockets amenable to selective targeting. Similarly, atypical chemokine receptors (ACKR1–ACKR5), although structurally classified as GPCRs, contain mutations at canonical G protein coupling motifs, rendering them incapable of signal transduction via traditional G protein pathways. These features, readily identifiable through sequence alignment, suggest new avenues for developing “decoy” or “scavenger” therapeutic strategies that modulate chemokine availability rather than downstream signaling.

Finally, when combined with structural prediction techniques, sequence alignment serves as a foundation for homology modeling or machine learning-based 3D structure prediction (e.g., AlphaFold3) (Abramson et al., 2024). This allows detailed analysis of how non-conserved residues impact the spatial conformation of receptor–ligand complexes. Computational chemistry methods can further validate whether these residues contribute to ligand binding affinity, specificity, or induced conformational transitions, thereby directly linking sequence variation to functional divergence. Such integration of sequence and structural data offers a powerful framework for guiding the rational design of highly selective chemokine receptor modulators.

SBDD targeting GPCRs has evolved from traditional reliance on X-ray crystallography and nuclear magnetic resonance (NMR) to leverage cryo-EM and computational breakthroughs, revolutionizing therapeutic discovery. Historically, X-ray/NMR-derived structures of orthosteric sites enabled rational ligand design through molecular docking and virtual screening, optimizing compound affinity and selectivity. However, these methods struggled to resolve dynamic receptor conformations and explore allosteric sites, limiting mechanistic insights. Recent advances in cryo-EM now capture GPCRs in near-atomic resolution across functional states—particularly when complexed with G proteins or β-arrestin—unlocking structural blueprints for designing allosteric modulators and biased ligands. Concurrently, large-scale virtual screening accelerates hit identification, expanding the druggable GPCR landscape (Congreve et al., 2020).

**FIGURE 5:**
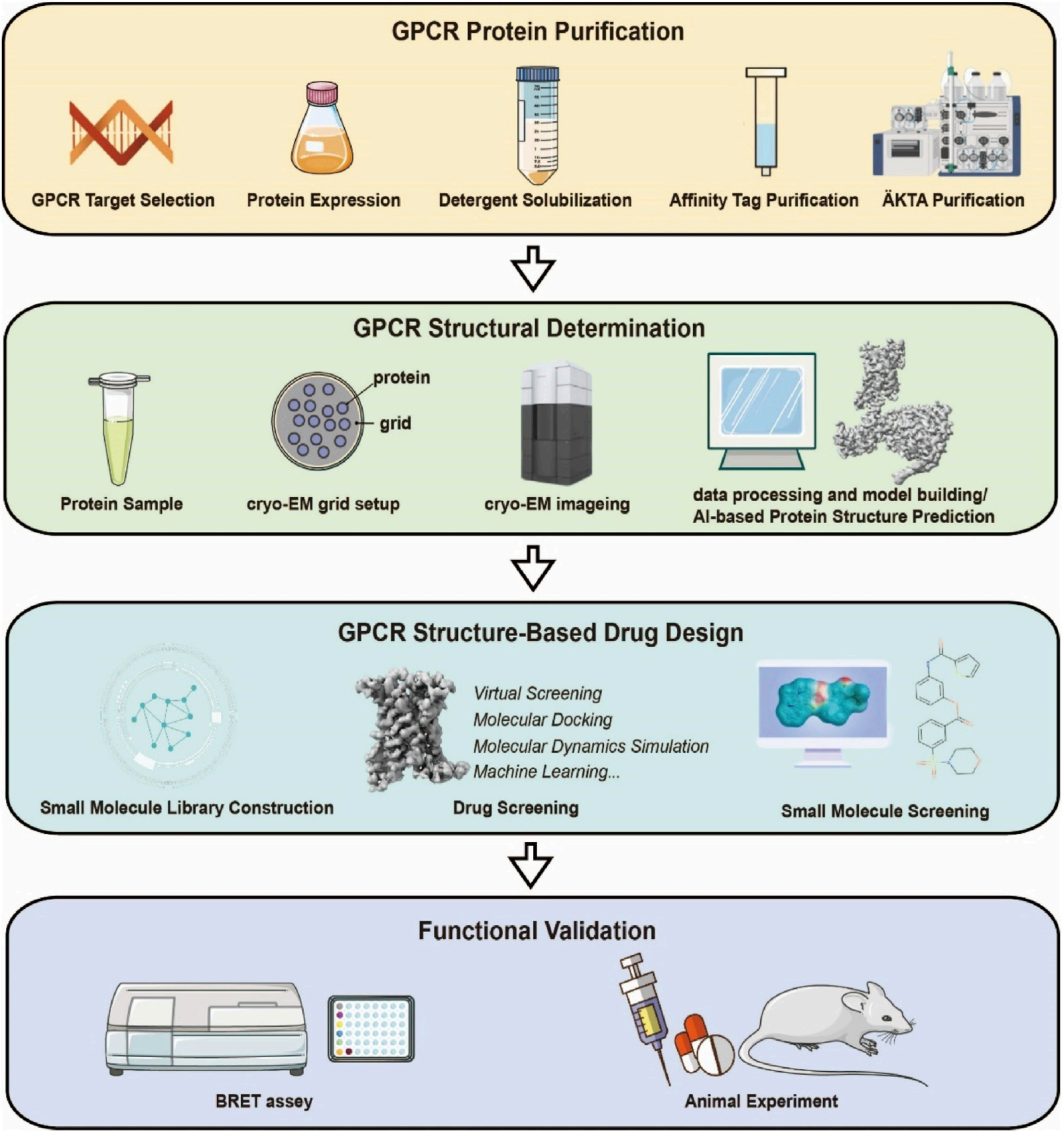
Research Workflow for Structure-Based Design of Small-Molecule Drugs Targeting GPCRs. The experimental workflow mainly consists of four parts: expression and purification of the target GPCR protein, cryo-EM sample preparation and structure determination, structure-based small-molecule screening, and functional validation.

Modern GPCR drug discovery integrates cryo-EM-derived structures with computational chemistry to drive precision. SBDD resolves ligand-bound receptor complexes (agonists, antagonists, allosteric modulators), mapping critical binding motifs to guide virtual screening and molecular docking. This structure-informed approach identifies novel chemotypes and optimizes existing scaffolds for enhanced affinity/selectivity prior to experimental validation. Compared to traditional methods, SBDD’s atomic-level insights enable pharmacophore refinement, accelerated fragment-based design, and biased agonist engineering to minimize off-target effects. By reducing screening randomness and streamlining lead optimization, SBDD shortens development timelines and lowers costs, transforming GPCR drug discovery (Duan J. et al., 2024) (Figure 5).

Structural insights into GPCRs have catalyzed precision drug discovery. Analysis of Adenosine A2A Receptor (A2AR) uncovered an underexplored hydrophobic subpocket, enabling the design of AZD4635—a highly selective antagonist with immuno-oncology potential (Marshall and Djamgoz, 2018). Fragment-based drug design (FBDD) synergized with X-ray data accelerates lead optimization, exemplified by HTL0014242, a negative allosteric modulator targeting Metabotropic Glutamate Receptor 5 (mGlu5) (Christopher et al., 2015). To address GPCR polymorphism and dynamics, mini-G proteins and nanobodies stabilize active receptor conformations, facilitating structural resolution of GPCR-G protein/β-arrestin complexes and elucidating signaling mechanisms.

Recent GPCR drug development advances highlight novel therapeutic paradigms. Allosteric modulators, binding non-orthosteric sites, fine-tune signaling with enhanced selectivity, as demonstrated by Metabotropic Glutamate Receptor (mGluR) and Muscarinic Acetylcholine Receptor M4 (M4 mAChR) modulators in neuropsychiatric trials (Orgován et al., 2019). Biased ligands decouple downstream pathways: oliceridine, a μ-opioid receptor agonist, retains analgesia while mitigating respiratory depression via selective G protein activation (Kenakin, 2019). Biologics like monoclonal antibodies/nanobodies expand GPCR targeting, with Glucagon-Like Peptide-1 (GLP-1) agonist/Gastric Inhibitory Polypeptide (GIP) antagonist combos showing efficacy against obesity/diabetes (Gutgesell et al., 2024). Drug repurposing strategies leverage existing pharmacotherapies: the β-blocker propranolol now treats infantile hemangiomas (Léauté-Labrèze et al., 2008) and cancer (Lin et al., 2023), shortening development timelines and de-risking translation (Lorente et al., 2025).

### 4.2 Advances in drug development targeting chemokine receptors

Targeting chemokine receptors represents a frontier in biomedical research, offering transformative therapeutic opportunities alongside persistent challenges. Advances in cryo-EM have decrypted high-resolution architectures of chemokine receptor complexes, unlocking ligand-binding architectures and critical residue networks. These insights provide critical molecular insights for small-molecule optimization and high-throughput drug screening. Allosteric antagonists, emerging as a strategic focus, surpass traditional orthosteric inhibitors by modulating receptor conformations, enhancing selectivity, and enabling biased signaling regulation—overcoming limitations in treating inflammation, cancer, and immune dysregulation.

**FIGURE 6:**
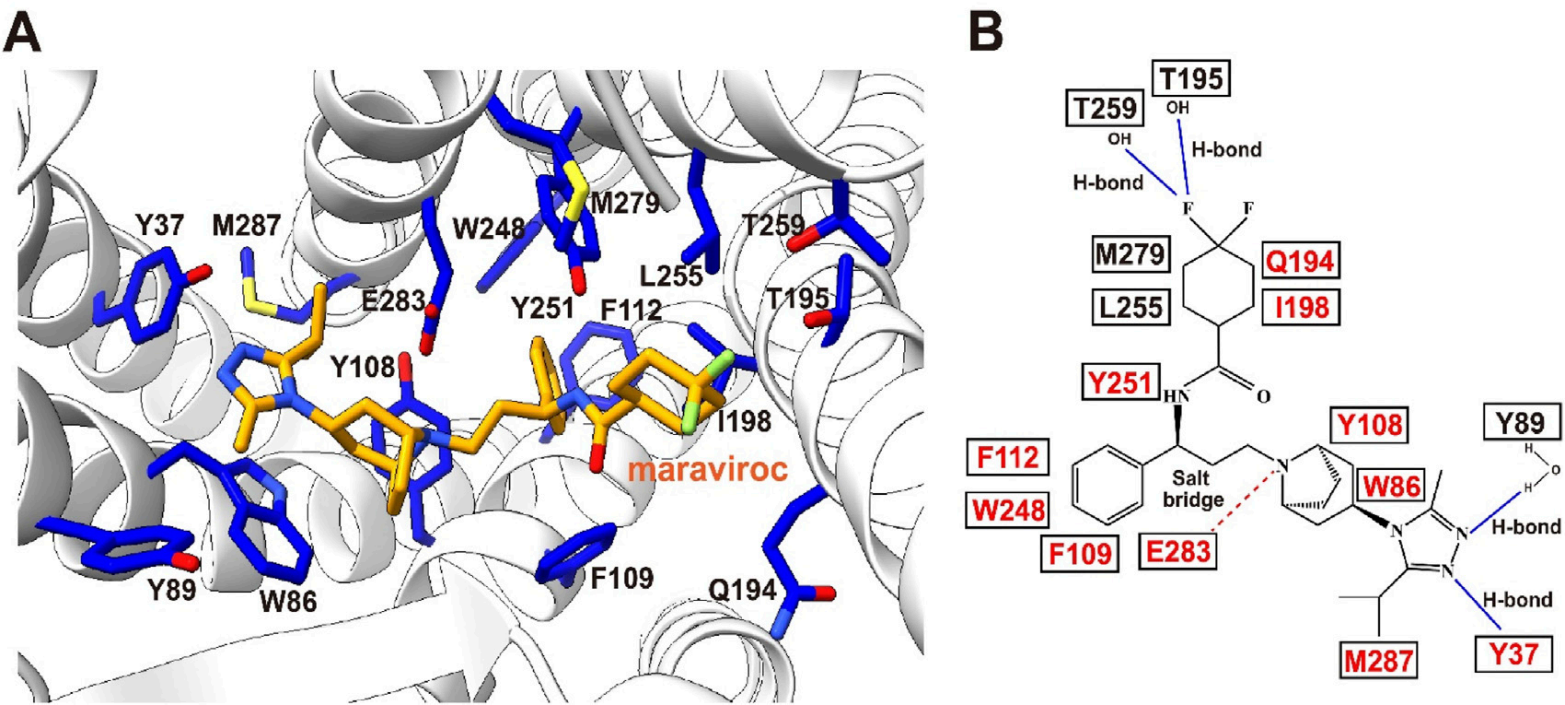
Maraviroc-binding pocket of CCR5. **(A)** Key residues involved in the binding of CCR5 to maraviroc. Maraviroc (orange) and interacting receptor residues (blue) are shown as sticks. **(B)** Schematic representation of the interactions between CCR5 and maraviroc. Mutations reported to be critical for maraviroc binding are highlighted in red. Maraviroc–CCR5 complex PDB: 4MBS.

The CCR5 structural paradigm has redefined anti-HIV drug design. Cryo-EM maps of CCR5 reveal its ligand-binding pocket topology, guiding the development of maraviroc, an allosteric inhibitor that occupies a deep TMD cavity formed by TM1–3, TM5–7. Maraviroc anchors via hydrogen bonds (E283, Y251) and hydrophobic contacts (Y108, F109, W248), stabilizing CCR5’s inactive state to block HIV-1 entry (Tan et al., 2013). Comparative structural analyses highlight divergent ligand-binding mechanisms between CCR5 and CXCR4. While both receptors share global folds, CCR5’s more accessible pocket and distinct charge distribution contrast with CXCR4’s N-terminus/ECL2-shielded cavity and acidic N-terminal residues, aligning with HIV-1 V3 loop tropism. These differences rationalize co-receptor specificity and inform allosteric inhibition strategies (Figure 6).

Extending these principles, CCR9’s structural resolution uncovers an intracellular allosteric site. Vercirnon engages this pocket by interacting with TM1–3, TM6–7, and H8, locking CCR9 in a G protein-uncoupled state. This intracellular antagonism mechanism diverges from classical GPCR ligand binding modes, broadening therapeutic design paradigms (Oswald et al., 2016).

Collectively, chemokine receptor structural biology delivers molecular blueprints for precision drug design, illuminating ligand engagement logic and accelerating therapies for HIV, immune disorders, and beyond (Table 2).

**TABLE 2:** Approved/clinical trial drugs targeting chemokine receptors.

Target	Drug	Development status	Mechanism of action	Therapeutic indication	Clinical trial phase/Market approval date	References
CCR1	AZD-4818	Investigational	Antagonist	Asthma and Chronic Obstructive Pulmonary Disease (COPD)	II	Norman (2009)
	BMS-817399	Investigational	Modulator	Rheumatoid Arthritis	II	Santella et al. (2014)
	CCX-354	Investigational	Antagonist	Rheumatoid Arthritis	II	Tak et al. (2013)
	MLN3897	Investigational	Antagonist	Rheumatoid Arthritis	II	Vergunst et al. (2009)
CCR2	BMS-813160	Investigational	Modulator	Pancreatic Ductal Adenocarcinoma	II	Cherney et al. (2021)
	CCX915	Investigational	Antagonist	Gonorrhea, Chlamydia, and Prostate Cancer	Ⅰ	Noman et al. (2023)
	Cenicriviroc	Investigational	Inhibitor	Non-Alcoholic Steatohepatitis (NASH)	III	Anstee et al. (2024)
	CCX140-B	Investigational	Antagonist	Type 2 Diabetes	II	de Zeeuw et al. (2015)
	INCB3284	Investigational	Antagonist	Hemorrhagic Shock	Ⅰ	DeSantis et al. (2022)
	MK-0812	Investigational	Antagonist	Diabetic Nephropathy	II	O'Brien et al. (2017)
	Pimagedine	Investigational	Antagonist	Diabetic Nephropathy	III	Abdel-Rahman and Bolton (2002)
	Plozalizumab	Investigational	Antagonist	Atherosclerosis	Ⅰ	Gilbert et al. (2011)
CCR3	ALK-4290	Investigational	Inhibitor	Age-Related Macular Degeneration (AMD)	II	Samanta et al. (2020)
CCR4	FLX475	Investigational	Antagonist	Tumors	II	Jorapur et al. (2022)
	Mogamulizumab	Approved	Antagonist	Mycosis Fungoides (MF) and Sézary Syndrome (SS)	2018.8	Lewis and Rook (2020)
CCR5	Aplaviroc	Investigational	Antagonist	HIV	III	Demarest et al. (2008)
	AZD-5672	Investigational	Antagonist	Neuropathic Pain	II	Cherney et al. (2021)
	BMS-813160	Investigational	Modulator	Pancreatic Ductal Adenocarcinoma	II	Ciechanowska et al. (2023)
CCR5	PRO 140	Investigational	Antagonist	HIV	III	Thompson (2018)
	Cenicriviroc	Investigational	Modulator	Non-Alcoholic Fatty Liver Disease (NAFLD)	III	Anstee et al. (2024)
	Ibalizumab	Approved	Antagonist	HIV	2018.03	Beccari et al. (2019)
	INCB-9471	Investigational	Antagonist	HIV	II	Brown et al. (2009)
	Leronlimab	Investigational	Antagonist	Breast Cancer; COVID-19	III	Jiao et al. (2021), Agresti et al. (2021)
	Maraviroc	Approved	Antagonist; Inhibitor	HIV	2007.08	Lieberman-Blum et al. (2008)
	PF-232798	Investigational	Antagonist	HIV	II	Zhu et al. (2019)
	Vicriviroc	Investigational	Antagonist	HIV	III	Klibanov (2009)
CCR9	Vercirnon	Investigational	Modulator	Crohn’s Disease	II	Arseneau and Cominelli (2013)
CXCR1	Ibuprofen	Approved	Inhibitor	Lipid Metabolism	1969	Cao et al. (2024)
	Ketoprofen	Approved, Veterinary Approved	Other	Rheumatoid Arthritis, Osteoarthritis, Ankylosing Spondylitis, Dysmenorrhea, Mild to Moderate Muscle Pain, Postoperative Pain, Postpartum Pain	1980	Kantor (1986)
	Navarixin	Investigational	Antagonist	Tumors	II	Jaeger et al. (2019)
	Reparixin	Investigational	Modulator	Myelofibrosis	III	Verachi et al. (2022)
	SX-682	Investigational	Inhibitor	Head and Neck Squamous Cell Carcinoma	II	Greene et al. (2020)
CXCR2	AZD-5069	Investigational	Inhibitor	Pancreatic Cancer	II	Shao et al. (2024)
	Clotrimazole	Approved, Veterinary Approved	Modulator	Fungal and Yeast Infections	1975.02	Sawyer et al. (1975)
	Danirixin	Investigational	Modulator	Breast Cancer	II	Nie et al. (2021)
	Ibuprofen	Approved	Inhibitor	Lipid Metabolism	1969	Cao et al. (2024)
	Navarixin	Investigational	Modulator	Tumors	II	Jaeger et al. (2019)
	Reparixin	Investigational	Modulator	Myelofibrosis	III	Verachi et al. (2022)
	SX-682	Investigational	Inhibitor	Head and Neck Squamous Cell Carcinoma	II	Greene et al. (2020)
	Tallimustine	Experimental	Antagonist	Leukemia	No data available	Beran et al. (1997)
CXCR4	Baclofen	Approved	Allosteric Modulator	Breast Cancer	1977	Guyon et al. (2013)
	Balixafortide	Investigational	Antagonist	Prostate Cancer	III	Robinson et al. (2023)
	Burixafor	Investigational	Inhibitor	Multiple Myeloma, Hodgkin’s Disease, Non-Hodgkin’s Lymphoma	II	Sukhtankar et al. (2023)
	CTCE-9908	Investigational	Modulator	Prostate Cancer, Breast Cancer, Esophageal Cancer	II	Wong et al. (2014), Huan et al. (2009), Drenckhan et al. (2013)
	Framycetin	Approved	Antagonist	Bacterial Blepharitis, Bacterial Enteritis, Corneal Injury, Corneal Ulcer, Meibomianitis	1952	Borkow et al. (2003)
	Ibalizumab	Approved	Antagonist	HIV	2018.03	Beccari et al. (2019)
	Mavorixafor	Approved	Antagonist; Inhibitor	WHIM Syndrome	2024.04	Hoy (2024)
	Motixafortide	Approved	Antagonist; Inhibitor	Pancreatic Cancer	2023.9	Hoy (2023)
	MSX-122	Investigational	Antagonist; Partial Antagonist	Cancer	Ⅰ	Yang et al. (2024)
	Plerixafor	Approved	Antagonist; Inhibitor	Non-Hodgkin’s Lymphoma, Multiple Myeloma	2008.12	Stewart et al. (2009)
	Ulocuplumab	Investigational	Inhibitor	Leukemia	II	Korbecki et al. (2024)
	USL-311	Investigational	Antagonist	Glioblastoma	II	Bao S et al. (2023)
CX3CR1	KAND567	Investigational	Inhibitor	Myocardial Infarction, Leukemia	II	Zhong et al. (2024)

Certain data were obtained from the DrugBank database (Knox et al., 2024).

## 5 Discussion

The development of chemokine receptor-targeted drugs faces significant translational challenges, with only a handful of approved antagonists and numerous candidates remaining in clinical trials or discontinued due to efficacy/safety concerns. Key obstacles include: 1) Poor drug selectivity stemming from high sequence homology among chemokine receptor family members, limiting single-target inhibition efficacy; 2) Signal redundancy within the complex chemokine network, where single ligands activate multiple receptors and vice versa, enabling compensatory signaling pathways; 3) Structural knowledge gaps for approximately 40% of chemokine receptors, hindering rational drug design; and 4) Clinical trial complexities including biomarker ambiguity and heterogeneous patient responses that complicate therapeutic validation.

By integrating cryo-EM, computational chemistry, and artificial intelligence (AI) technologies, researchers can further reveal the dynamic conformational changes of receptors in different functional states. Combining structural biology and functional studies, the development of highly selective allosteric antagonists, biased ligands, and therapeutic antibodies holds promise for achieving precise interventions targeting specific receptor subtypes or signaling pathways. With the resolution of more chemokine receptor structures and their complexes, coupled with AI-driven molecular design, future advancements may revolutionize the treatment of inflammatory diseases, autoimmune disorders, and cancer, providing new theoretical foundations and technical support for drug development. Additionally, given the complex regulation of the chemokine network, targeting multiple chemokines or their receptors simultaneously may offer more effective therapeutic strategies. In summary, chemokine receptor drug development is a field full of challenges but also opportunities. We aim to summarize the latest advances in chemokine receptor-targeted therapies and inspire researchers to drive progress in this field. Future research should focus on developing more effective, selective, and safer small-molecule drugs, addressing challenges such as receptor redundancy and clinical translation barriers. By integrating emerging technologies like AI-driven drug design and advanced structural biology, this field has the potential to provide transformative treatments for cancer, chronic inflammatory diseases, and beyond.

## Supplementary material

SUPPLEMENTARY FIGURE S1 Sequence alignment of human chemokine receptors reveals conserved structural motifs and selectivity-determining regions. This figure presents a multiple sequence alignment of human CCR, CXCR, XCR, CX3CR, and ACKR family members. Conserved GPCR motifs—such as DRY, CWxP, and NPxxY—are retained across transmembrane domains, indicating their essential roles in signaling. In contrast, notable sequence divergence is observed in the N-terminal and ECL2 regions, which likely contribute to ligand specificity and offer potential targets for selective drug design.
