# Supplementary material for: Progress in structure-based drug development targeting chemokine receptors
Source: Front Pharmacol. 2025 Jun 9;16:1603950. doi: 10.3389/fphar.2025.1603950 (PMC12183272; doi:10.3389/fphar.2025.1603950)
Supplement: Supplementary file 1 [file Image1.pdf]

```
                                    10
CCRI     ......METPNTTEDYDTT. .................. TEFDYGDATP.      QK
CCR2       ....MLSTSRSRFIRNTNE. ...........SGEEVTTFFDYDYGAP.      HK
CCR3       ....MTTSLDTVETFGTT.. ................. SYYDD.VGLL.      EK
CCR4     ............MNPTDIADTTLDESI.         ...YSN ... YYLYESIPKP.      TK
CCRS     .............MDYQVSSPIYDIN... ..................... YYTSEP.      QK
CCR6     ............MSGESMNFSDVFDSS.         ...EDYFVSVNTSYYSVDSEMLL SL
CCR?     MDLGKPMKSVLVVALLVIFQVCLCQDE.         ...VTDDYIGDNTTVDYTLFESL SK
CCRS     ............MDYTLDLSVTTVTD... ................ YYYPDIFSSP.      DA
CCR9     ......MTPTDFTSPIPN....MADDY..........GSESTSSMEDYVNFNFTDFY  EK
CCRIO       ...MGTEATEQVSWG.                 ..HYSGDEEDAYSAEPLPEL  YK
CXCRI     ..............MSNITDPQMWDFD..........DLN....FTGMPPADEDYSP ML
CXCR2     .........MEDFNMESDSFEDFWKGE.        ...DLSNYSYSSTLPPFLLDAAP EP
CXCR3         .MVLEVSDHQVLNDAEVAALLENFSSSYDYGENESDSCCTSPP.      PQ
CXCR4       ......MEGISIYTSDNYTEEM..........G........ SGDYDSMKEP.      FR
CXCRS        ...MNYPLTLEMDLEN.     .LEDLFWELDRLDNYNDTSLVENHLCPA EG
CXCR6                     .MAEH.    ......DYHEDYGFSSFNDSSQEE........H
XCRI     ............MESSGNP ....................ESTTFFYYDLQSQP ........ CE
CX3CR1          ..MDQFPESVTEN. ................... FEYDDLAEA.      YI
ACKRI    ...MGNCLHRAELSPSTENSSQLDFEDVWN.......SSYGVNDSFPDGDYGANLEA  AP
ACKR2    ............MAATASPQPLATEDA..........DSENSSFYYYDYLDEVAFML  RK
ACKR3    ............MDLHLFDYSEPGNFS............DISWPCNSSDCIVVDTVM  PN
ACKR4    ............MALEQN ....QSTDY ..........YYEENE.MNGTYDYSQYELI  IK
ACKRS        ....MANYTLAPEDEYDVLI.         ...EG ..... ELESDEAEQ.      DK
```

```
            3□                                              70        80
CCRI     VNERAFGAQ                                    YKRLKNMTSIYLLNLAISDLLFL
CCR2     FDVKQIGAQ                                    CKKLKCLTDIYLLNLAISDLLFL
CCR3     ADTRALMAQ                                    YRRLRIMTNIYLLNLAISDLLFL
CCR4     EGIKAFGEL                                   .                     FV
CCRS     INVKQIAAR                                   .                     FL
CCR6     QEVRQFSRL                                   .                     FV
CCR?     KDVRNFKAW                                  ..                     FL
CCRS     ELIQTNGKL                                  ..                     FV
CCR9     NNVRQFASH                                  ..                     FL
CCRIO    ADVQAFSRA                                  A.                     LA
CXCRI    .ETETLNKY                                  ..                     FA
CXCR2    .ESLEINKY                                  .                      FA
CXCR3    DFSLNFDRA                                  .                      LV
CXCR4    EENANFNKI                                  .                      FV
CXCRS    PLMASFKAV                                  R.                      V
CXCR6    QDFLQFSKV                                  ..                     FV
XCRI     NQAWVFATL                                  ..                     FA
CX3CR1   GDIVVFGTV                                  ..                     FV
ACKRI    CHSCNLLDD                                  PL                     FS
ACKR2    DAVVSFGKV                                  ..                     FL
ACKR3    MPNKSVLLYTLSFIY                            ..AKTTGYDTHCYILNLAIADLWVV
ACKR4    EDVREFAKV                                  ..YKKQRTKTDVYILNLAVADLLLL
ACKRS    YDAQALSAQ LVPSLCSAVFVIGVLDNLLVVLILVK..YKGLKRVENIYLLNLAVSNLCFL
```

```
              90        100       110                  3            14
CCRI     FTLPFWIDYKLKD.DWVFGDAMCKILSGFYYTGL::: IFF I IL[illegible]I RYIAI   vHAVFAL.
CCR2     ITLPLWAHSAAN..EWVFGNAMCKLFTGLYHIGYFGGIFFIILLTIDRYLAI VHAVFAL.
CCR3     VTLPFWIHYVRGH.NWVFGHGMCKLLSGFYHTGLYSEIFFIILLTIDRYLAI VHAVFAL.
CCR4     FSLPFWGYYAAD..QWVFGLGLCKMISWMYLVGFYSGIFF ML SIDRYLAIVHAVFSL.
CCRS     LTVPFWAHYAAA..QWDFGNTMCQLLTGLYFIGFFSGIFFIILLTIDRYLAV VHAVFAL.
CCR6     LTLPFWAVSHATG.AWVFSNATCKLLKGIYAINF CG LLLTCISMDRYIAIVQATKSFR
CCR?     LTLPFWAYSAAKS..WVFGVHFCKLIFAIYKMSF SG LLLLCISIDRYVAIVQAVSAHR
CCRS     FSFPFQTYYLLD..QWVFGTVMCKVVSGFYYIGFYSS  FFIT  SVDRYLAVVHAVYAL.
CCR9     VTLPFWAIAAADQ..WKFQTFMCKVVNSMYKMNFYSC  LLIM  SVDRYIAIAQAMRAHT
CCRIO    LTLPFAAAGALQG..WSLGSATCRTISGLYSASFHAGFLFLA  SADRYVAIARALPAG.
CXCRI    LTLPIWAASKVNG..WIFGTFLCKVVSLLKEVNFYSGILLLA  SVDRYLAIVHATRTLT
CXCR2    LTLPIWAASKVNG..WIFGTFLCKVVSLLKEVNFYSGILLLA  SVDRYLAIVHATRTLT
CXCR3    LTLPLWAVDAAVQ..WVFGSGLCKVAGALFNINFYAG  LLLA  SFDRYLNIVHATQLY.
CXCR4    ITLPFWAVDAVA..NWYFGNFLCKAVHVIYTVNLYSS  LILA  SLDRYLAIVHATNSQ.
CXCRS    FILPFAVAEGSVG..WVLGTFLCKTVIALHKVNFYCSSLLLA  AVDRYLAIVHAVHAY.
CXCR6    CTLPFWAYAGIHE..WVFGQVMCKSLLGIYTINFYTS  LILT  TVDRFIVVVKATKAYN
XCRI     CLLPVWISPYHW..GWVLGDFLCKLLNMIFSISLYSSIFFLT  TIHRYLSVVSPLSTL.
CX3CR1   ATLPFWTHYLIN..EKGLHNAMCKFTTAFFFIGFFGSIFFIT  SIDRYLAIVLAANSM.
ACKRI    IVVPVLAPGLGST....RSSALCSLGYCVWYGSAFAQ  LLLG  ASLGHRLGAGQVPGL.
ACKR2    VTLPFWGISVAW..HWVFGSFLCKMVSTLYTINFYSGIFFIS  SLDKYLEIVHAQPYH.
ACKR3    LTIPVWVVSLVQHNQWPMGELTCKVTHLIFSINLFGSIFFLT  SVDRYLSITYFTNTP.
ACKR4    FTLPFWAVNAVHG..WVLGKIMCKITSALYTLNF SG QFLACISIDRYVAVTKVPSQSG
ACKRS    LTLPFWAH........AGGDPMCKILIGLYFVGLYSETFF CLLTV RYLVFLHKGNFF.
```

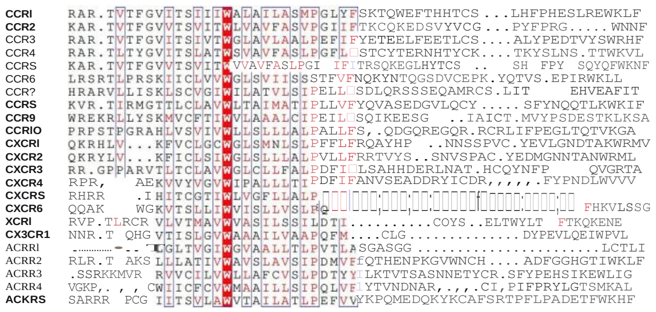

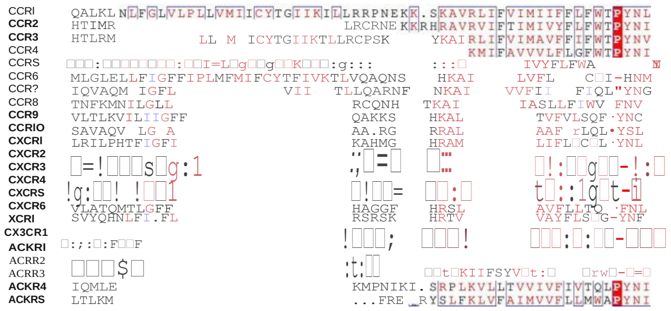

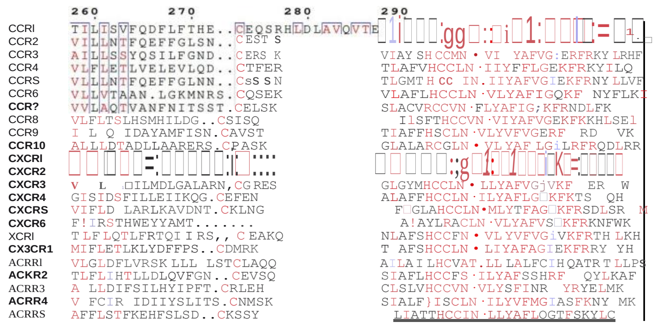

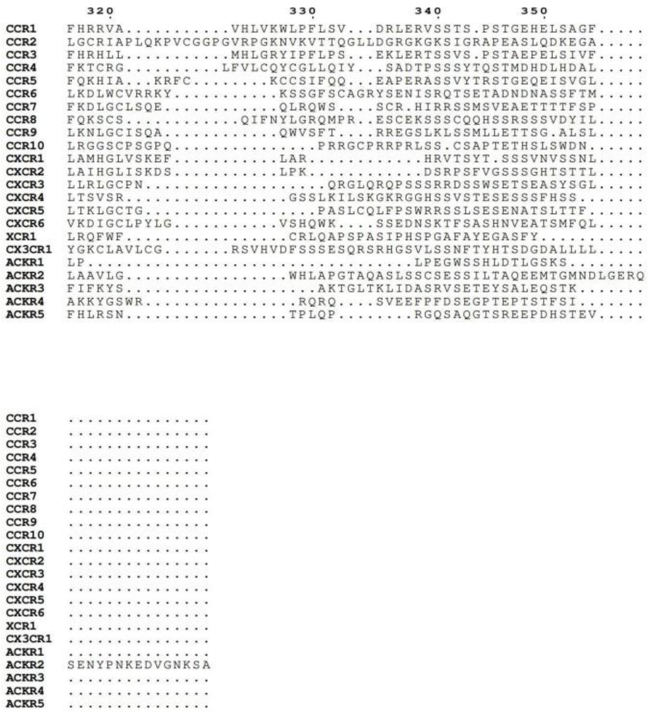

Figure S1. Sequence alignment of human chemokine receptors reveals conserved structural motifs and selectivity-determining regions. This figure presents a multiple sequence alignment of human CCR, CXCR, XCR, CX3CR, and ACKR family members. Conserved GPCR motifs—such as DRY, CWxP, and NPxxY—are retained across transmembrane domains, indicating their essential roles in signaling. In contrast, notable sequence divergence is observed in the N-terminal and ECL2 regions, which likely contribute to ligand specificity and offer potential targets for selective drug design.
